# Urinary Creatinine Concentrations in the U.S. Population: Implications for Urinary Biologic Monitoring Measurements

**DOI:** 10.1289/ehp.7337

**Published:** 2004-09-23

**Authors:** Dana B. Barr, Lynn C. Wilder, Samuel P. Caudill, Amanda J. Gonzalez, Lance L. Needham, James L. Pirkle

**Affiliations:** ^1^National Center for Environmental Health, Centers for Disease Control and Prevention, Atlanta, Georgia, USA; ^2^Agency for Toxic Substances and Disease Registry, Atlanta, Georgia, USA

**Keywords:** biomonitoring, creatinine, creatinine adjustment, urine

## Abstract

Biologic monitoring (i.e., biomonitoring) is used to assess human exposures to environmental and workplace chemicals. Urinary biomonitoring data typically are adjusted to a constant creatinine concentration to correct for variable dilutions among spot samples. Traditionally, this approach has been used in population groups without much diversity. The inclusion of multiple demographic groups in studies using biomonitoring for exposure assessment has increased the variability in the urinary creatinine levels in these study populations. Our objectives were to document the normal range of urinary creatinine concentrations among various demographic groups, evaluate the impact that variations in creatinine concentrations can have on classifying exposure status of individuals in epidemiologic studies, and recommend an approach using multiple regression to adjust for variations in creatinine in multivariate analyses. We performed a weighted multivariate analysis of urinary creatinine concentrations in 22,245 participants of the Third National Health and Nutrition Examination Survey (1988–1994) and established reference ranges (10th–90th percentiles) for each demographic and age category. Significant predictors of urinary creatinine concentration included age group, sex, race/ethnicity, body mass index, and fat-free mass. Time of day that urine samples were collected made a small but statistically significant difference in creatinine concentrations. For an individual, the creatinine-adjusted concentration of an analyte should be compared with a “reference” range derived from persons in a similar demographic group (e.g., children with children, adults with adults). For multiple regression analysis of population groups, we recommend that the analyte concentration (unadjusted for creatinine) should be included in the analysis with urinary creatinine added as a separate independent variable. This approach allows the urinary analyte concentration to be appropriately adjusted for urinary creatinine and the statistical significance of other variables in the model to be independent of effects of creatinine concentration.

Biologic monitoring (i.e., biomonitoring) is used to assess human exposures to environmental and workplace chemicals. The most commonly used matrices for biomonitoring are blood (and its components, e.g., serum and plasma) and urine. The average blood volume of an individual changes an average of 80 mL/kg body weight (Guyton and Hall 2000) and remains relatively constant for a healthy individual who maintains a given body weight; thus, changes in blood concentrations of selected environmental and workplace chemicals in individuals or populations can be readily evaluated. For example, in the Third National Health and Nutrition Examination Survey (NHANES III) (Brody et al. 1994), blood lead concentrations demonstrated the decline in the concentrations of lead in the U.S. population between the Second NHANES (NHANES II), 1976–1980, and the first phase of NHANES III, 1988–1991 (Pirkle et al. 1994). Blood has also been used to evaluate exposures to lipophilic compounds, such as polychlorinated dibenzo-*p*-dioxins, polychlorinated biphenyls, and organochlorine insecticides. These chemicals are reported in blood and serum based on their lipid content, which varies among individuals and within an individual, especially after eating. Adjusting based upon lipid content allows direct comparisons of their concentrations within and among individuals, regardless of the amount of lipid in the blood, and also comparisons among various biologic matrices, such as blood and adipose tissue (Phillips et al. 1989).

Urine also is a widely used matrix for biomonitoring, especially for nonpersistent chemicals (i.e., chemicals that have short biologic half-lives), such as some current-use pesticides, metals, and drugs. One of the major advantages of using urine in biomonitoring is its ease of collection for spot or grab (untimed) urine samples but not for 24-hr urine voids, because 24-hr collection can be cumbersome, often resulting in improper or incomplete collection. Therefore, spot urine samples, whether first-morning voids or “convenience” samples, are generally used for biomonitoring. The major disadvantages of spot urine samples include the variability in the volume of urine and the concentrations of endogenous and exogenous chemicals from void to void. How to best adjust the urinary concentrations of environmental chemicals in a manner analogous to the adjustment of the concentrations of lipophilic chemicals in blood samples remains a subject of research.

Variations in urinary analyte concentrations from changing water content in urine have been eliminated using urinary excretion rate (UER) calculations (Rigas et al. 2001). To calculate the UER, the metabolite concentration in urine is multiplied by the volume of the void and then divided by the duration of time the void was accumulating in the bladder. This model assumes that the entire bladder is emptied with each void and that the entire sampling void volume is known. Because this is based on the mass in the sample, variability in urine concentrations from urine dilution is removed, particularly for analytes where the rate of excretion varies with the urine flow (Boeniger et al. 1993). However, because the void volume and times of previous and current voids are required, this approach is often not practical for epidemiologic studies, especially those studies involving young children or large population groups.

Urinary creatinine concentrations, specific gravity, and osmolality are common methods for adjusting dilution and for determining whether a spot urine sample is valid for assessing chemical exposures. The most widely used method is creatinine adjustment that involves dividing the analyte concentration (micrograms analyte per liter urine) by the creatinine concentration (grams creatinine per liter urine). Analyte results are then reported as weight of analyte per gram of creatinine (micrograms analyte per gram creatinine).

Many studies have documented that creatinine-adjusted urinary metabolite concentrations correlate better with blood, serum, or plasma concentrations of the parent chemical than the unadjusted concentrations, suggesting that creatinine-adjusted analyte concentrations may serve as good surrogates for size-related dose (Cline et al. 1989; Hill et al. 1995a; Shealy et al. 1997; To-Figueras et al. 1997). However, these studies typically self-correct for size variation because each data pair is from a single individual. Thus, children, who have blood volumes that are proportionately smaller (80 mL less per kilogram body weight), would have higher blood concentrations of a chemical after the absorption of the same amount of chemical after an exposure compared with adults with an identical exposure. Similarly, their lower urinary creatinine concentrations would increase the creatinine-adjusted urine concentration of the metabolite compared with that of an adult with an identical exposure. Therefore, the paired urine and blood values from children and adults can be easily used to determine the relationship between matrices within an individual, but this does not necessarily mean that the creatinine-adjusted metabolite concentrations can be used to accurately compare exposures among the study participants.

Creatinine concentrations also are used to determine whether the spot urinary sample is valid. The guidelines of the World Health Organization (WHO) for valid urine samples for occupational monitoring often are used. The WHO recommends that if a sample is too dilute (creatinine concentration < 30 mg/dL) or too concentrated (creatinine concentration > 300 mg/dL), another urine void should be collected (WHO 1996) and analyzed for creatinine and the target chemical. These guidelines have been adopted for biomonitoring in the U.S. workplace (Lauwerys and Hoet 1993). The U.S. Department of Transportation defines an acceptable urine specimen for the screening of selected drugs of abuse as one that has a creatinine concentration of ≥5 mg/dL and a specific gravity of 1.001–1.020 (Barbanel et al. 2002). Urine of “normal” persons would be unlikely to be excluded using these criteria (Barbanel et al. 2002).

Urine creatinine concentrations were used to adjust the urinary concentrations of pesticides and metabolites of pesticides and phthalates in subsets of adults participating in NHANES III. These “creatinine-corrected” concentrations (micrograms analyte per gram creatinine) were reported in addition to the unadjusted concentrations in micrograms analyte per liter urine (Blount et al. 2000; Hill et al. 1995b). These reports also used the WHO’s recommendation for exclusion of samples, regardless of age (these were all adults), sex, or race/ethnicity.

Because urinary creatinine concentrations are so widely used to adjust or correct urinary concentrations of environmental and workplace chemicals or their metabolites, the formation of urinary creatinine and the ways in which various factors may affect its concentration are important to review. Creatinine is a waste product formed by the spontaneous, essentially irreversible dehydration of body creatine and creatine phosphate from muscle metabolism. A total of 94–98% of total creatine is accumulated within skeletal muscle. The rate of creatinine formation is fairly constant, with approximately 2% of body creatine converted to creatinine every 24 hr; this rate decreases with age in adults.

Creatinine is cleared from the body through the kidney primarily by glomerular filtration. However, 15–20% of the creatinine in urine can occur by active secretion from the blood through the renal tubules (Boeniger et al. 1993). The rate of secretion can vary substantially among persons because of various genetic and biologic factors. Researchers have found a high correlation between urinary creatinine concentrations and muscle mass (Edwards and Whyte 1959; Fuller and Rich 1982); higher urinary creatinine concentrations in men than in women (Bjornsson 1979; Turner and Cohn 1975); decreased urinary creatinine concentrations in adults with increasing age, probably because of a general decline in muscularity and glomerular filtration rate (GFR) (Alessio et al. 1985; Drive and McAlevy 1980); and seasonal variation in creatinine concentrations in children (Freeman et al. 1995; O’Rourke et al. 2000). In addition, persons with a high red meat intake have a higher urinary creatinine concentration than do those on a low-red-meat diet (Lykken et al. 1980). The effects of these factors and others on urinary creatinine concentrations have been reviewed (Boeniger et al. 1993).

Because of the relatively constant excretion rate of creatinine into the urine (which makes urinary creatinine concentration inversely proportional to urine flow rate), creatinine adjustment has been used to normalize analyte concentrations in spot samples for occupational and environmental exposure monitoring. This approach reportedly works well for individual occupational exposure analysis (e.g., preshift and postshift samples from the same person) if the analyte measured behaves similarly to creatinine in the kidney (Boeniger et al. 1993). However, if the analyte is excreted predominantly through passive secretion in the kidney, the analyte secretion will vary with urine flow rate and creatinine adjustment would not correct for urine concentration/dilution.

Urinary creatinine concentration data have been used to adjust urinary concentrations of environmental and workplace chemicals, primarily in adults. Thus, most of the published urinary creatinine concentration data are for adults. However, as more emphasis is placed on children’s health issues and assessment of their exposures to environmental contaminants, biomonitoring of younger populations is also increasing (Needham and Sexton 2000; O’Fallon et al. 2000).

Our study objectives were to document the normal range of urinary creatinine concentrations among various demographic groups, evaluate the influence demographic variations in creatinine concentrations can have on biologic monitoring measurements, and explore methods to appropriately adjust urinary analytes using creatinine that take into account demographic differences in urinary creatinine levels. In this article, we present urinary creatinine concentrations in samples collected during 1988–1994 throughout the United States from NHANES III participants. We describe the distribution of urinary creatinine concentrations within this population by age, sex, and race/ethnicity for persons ≥6 years of age. We also examine other factors that can affect urinary creatinine concentrations, such as body mass index (BMI), fat-free mass (FFM), and health status: kidney function, hyperthyroidism, hypertension, and diabetes (Boeniger et al. 1993). In addition, we compare urinary creatinine concentrations in urine samples collected at three different times throughout the day (morning, afternoon, and early evening). Finally, we propose a multiple regression approach to adjusting urinary analytes for differences in creatinine concentration. This information will greatly assist researchers, occupational health physicians, risk assessors, public health officials, and other users of urinary biomonitoring data to better analyze and interpret urinary biomonitoring measurements.

## Materials and Methods

NHANES III, which was conducted by the National Center for Health Statistics (NCHS) of the Centers for Disease Control and Prevention (CDC), was a 6-year survey during 1988–1994 designed to measure the health and nutrition status of the civilian, noninstitutionalized U.S. population ≥2 months of age. National population estimates and estimates for the three largest racial/ethnic subgroups in the U.S. population (non-Hispanic white, non-Hispanic black, and Mexican American) can be derived from each of the two individual 3-year phases (1988–1991 and 1992–1994) and from the full 6-year survey.

Sampling selection for NHANES III was based on a complex multistage area probability design. Children younger than 5 years, adults ≥60 years of age, non-Hispanic blacks, and Mexican Americans were oversampled to allow an adequate number of sample persons in these demographic groups from which population-based estimates could be derived. However, urine samples were not collected for children < 6 years of age. Data were collected through a household interview, and a standardized physical examination was conducted in a mobile examination center. Urine specimens for analyses, including those for measuring creatinine concentrations, were collected during this examination throughout the day. Pre-examination procedures depended on the age and health status of the individual. For example, persons > 12 years of age were asked to fast for 2–12 hr, depending on appointment times, and persons with known diabetes or < 12 years of age were asked to eat a normal diet before the examination. Sociodemographic information and medical histories of the survey participants and their families were collected during the household interviews. Details of the sample design have been published (Ezzati et al. 1992). The data set used in our analysis is a part of the public release data set for NHANES III (NCHS 2004a).

### Laboratory methods

During the physical examinations, urine specimens were collected, stored cold (2–4°C) or frozen, and sent to the Fairview University Medical Center (Minneapolis, MN), where they were analyzed for creatinine using an automated colorimetric determination based on a modified Jaffe reaction using a Beckman Synchron AS/ASTRA clinical analyzer (Beckman Instruments, Brea, CA) (Jaffe 1886). The laboratory and method were certified according to guidelines set forth in the Clinical Laboratory Improvement Act and Amendment (1988).

### Demographic covariates

Age was reported at the time of the household interview as the age in years at the last birthday. Age categories used in our statistical analyses were 6–11, 12–19, 20–29, 30–39, 40–49, 50–59, 60–69, and ≥70 years. A composite racial/ethnic variable based on reported race and ethnicity was created to define three major racial/ethnic groups: non-Hispanic black, non-Hispanic white, and Mexican American. Persons who self-reported race as none of the three major racial/ethnic groups were included in the overall estimates but excluded from analyses in which race/ethnicity was the stratification variable.

### Health status definitions

The health status of participants was considered in the data analysis. All participants were tested for a variety of physical conditions that have been reported to potentially affect urinary creatinine concentrations. Participants were not screened for a given condition if they reported having been previously informed by a physician as having one of the conditions. Clinical parameters for determining the health status of individuals are summarized in Table 1.

The GFR used for kidney function analysis was calculated using the equation derived from the Modification of Diet in Renal Disease (MDRD) study (Coresh et al 2002, 2003; Levey et al. 2003), in which serum creatinine, age, sex, and race were used. Serum creatinine measurements for the MDRD study and the NHANES III study were performed in different laboratories, and a laboratory bias was observed (Coresh et al. 2002). Thus, serum creatinine values in the NHANES III data set were calibrated to be more comparable with the laboratory data obtained in the MDRD study by subtracting 0.23 mg/dL from each value (Coresh et al. 2002, 2003). For our analysis, we considered persons to have kidney dysfunction if their GFR was < 60 mL/min/1.73 m^2^, indicative of moderately or severely decreased kidney function (Levey et al. 2003).

### Statistical analysis

We analyzed data using the NHANES III analytic guidelines (NCHS 2004b) for sample size and coefficient of variation to ensure reliability of estimates. Survey-specific sample weights were used in statistical analyses. Arithmetic means, selected percentiles of urinary creatinine concentrations, and their respective confidence intervals were calculated using SAS release 8 (SAS Institute, Cary, NC) and the SUDAAN (release 7.5.6; Research Triangle Institute International, Research Triangle Park, NC) Proc Descript procedure. SUDAAN incorporates the NHANES III sampling weights and adjusts for the complex sample design of NHANES III. Sample weights account for the unequal probabilities of selection resulting from the cluster design and the planned oversampling of certain subgroups. Oversampling of children, the elderly, non-Hispanic blacks, and Mexican Americans necessitated the use of sampling weights in all analyses to produce national estimates of prevalence and associated variances. Because Proc Descript does not provide design effect estimates for distribution percentiles, we multiplied the design effect associated with a mean by 30 or 80 [i.e., the NCHS-recommended sample size for estimating a proportion of 0.50 (*n* = 30) or a proportion of 0.10 or 0.90 (*n* = 80) when the design effect is 1.0 (NCHS 2004b)]. If this product was larger than the actual sample size, we determined that the percentile estimate should not be reported. All distribution percentiles reported met this criterion.

The collective data set of urinary creatinine values was slightly skewed toward higher values; however, logarithmic transformation did not improve the shape of the distribution. Because the results were only slightly skewed and variance estimates obtained using SUDAAN software were robust, we chose not to transform the urinary creatinine results for the analysis.

An analysis of covariance was used to correct for demographic covariates before comparing concentrations among demographic groups and daily collection times. Statistical significance was set at *p* < 0.05.

Similar to the approach used by Wilder et al. (unpublished data), we used multiple linear regression models to study the influence of standard demographic variables on urinary creatinine concentration and additional factors previously reported to affect urinary creatinine concentrations. Nine variables were evaluated, although all variables were not used in the final model: race/ethnicity, sex, age, BMI, FFM, diabetes status, hypertension status, hyperthyroid disease status, and kidney disease status. FFM was calculated using a sex-and age-specific bioelectrical impedance analysis equation reported by Deurenberg et al. (1991). Height, weight, age, sex, and reactance measurements (ohms at 50 kHz) were used in the equation to derive individual estimates of FFM. Reactance measurements were available only for persons ≥12 years of age.

Our analysis comprised 22,245 valid creatinine values in urine samples collected during 1988–1994. Although we did not perform a thorough analysis of the rate of nonresponse and its possible effects on our analyses, we did evaluate the potential effects of differential nonresponse using the method of Flegal et al. (1991). We analyzed major demographic variables obtained from the interview data for persons with urinary creatinine values and persons without urinary creatinine values. For each variable, we compared the observed mean urinary creatinine level with the expected mean value for persons in the interviewed sample after we adjusted for that variable. The comparison assumed no statistical significance from differential nonresponse if the estimates were within 10% of the expected means (Flegal et al. 1991). We did not detect bias resulting from differential nonresponse for any of the previously listed variables.

## Results

**Figure 1:**
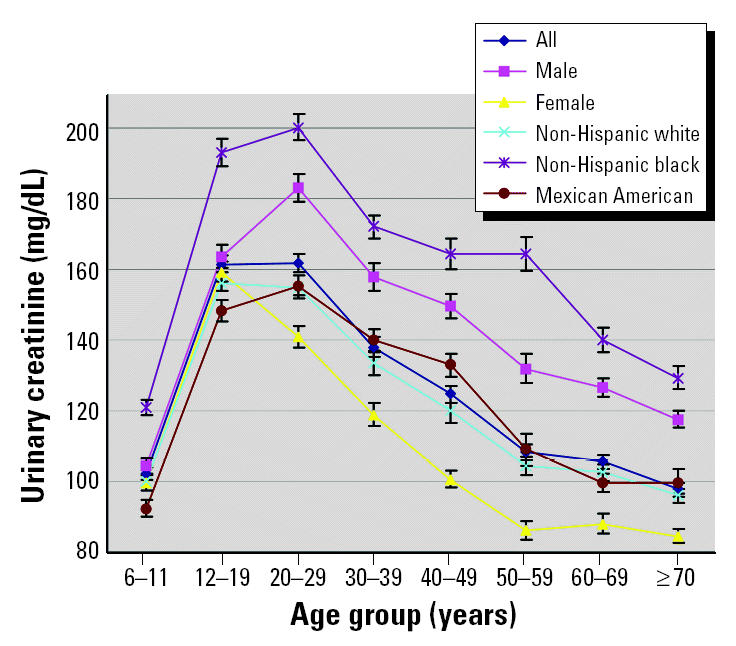
Mean urinary creatinine concentrations (mg/dL) for each sex and racial/ethnic group by age group.

The weighted urinary creatinine arithmetic means, medians, 10th and 90th percentiles, and their respective upper and lower 95% confidence intervals (CIs) are shown in Table 2. The data are shown both collectively and divided into age, race/ethnicity, and sex categories. No data were excluded from the distribution analysis. Non-Hispanic blacks had significantly greater concentrations of urinary creatinine than did all other racial/ethnic groups, across all age groups (*p* < 0.0006; Figure 1). On average, blacks had 33.43 and 34.25 mg more creatinine per deciliter of urine than did Mexican Americans and non-Hispanic whites, respectively. Adult (i.e., ≥20 years of age) males had significantly greater urinary creatinine than did adult females (*p* < 0.0001).

The percentage of individuals in each demographic group that had urinary creatinine concentrations outside the WHO exclusionary guidelines is shown in Table 3. Recently, Wilder et al. (unpublished data) reported that these exclusionary criteria should be re-evaluated for urine samples taken from children. In that study (410 children 1–8 years of age), 12% of all children fell below the guideline value, and 0% were too concentrated. Up to 8% of the NHANES samples examined had urinary creatinine concentrations < 30 mg/dL, whereas < 3% had concentrations > 300 mg/dL. Although these percentages differed for each demographic category, more samples were considered “too dilute” than “too concentrated.”

We did not have the information to classify the diabetic status or kidney function of persons 6–19 years of age; thus, we first limited our multiple linear regression analysis to subjects ≥20 years of age to determine the effects of diabetes and kidney function on urinary creatinine. For subjects ≥20 years of age, statistically significant categorical independent variables in the model included race/ethnicity, sex, diabetic status, kidney function status, and age group. The continuous independent variable BMI was also a statistically significant factor. There were statistically significant interactions between race and diabetic status (*p* = 0.0022), between race and kidney function status (*p* = 0.0073), between race and age group (*p* = 0.0028), between sex and age group (*p* = 0.0260), and between diabetic status and age group (*p* = 0.0133). Hyperthyroidism, hypertension, and FFM were not significant factors in the model and thus were not included in the final model.

Participants with diabetes tended to have lower urinary creatinine levels than did those without diabetes, and the magnitude of the decrease varied significantly among the three racial/ethnic groups studied and among the age categories. For example, non-Hispanic black participants with diabetes had urinary creatinine levels 34.2 mg/dL lower (*p* < 0.0001) than those without diabetes in the same ethnic group, whereas no significant differences were observed in the other racial/ethnic groups. Similar variation was observed for persons with diabetes in different age group categories. For example, urinary creatinine levels for persons with diabetes 30–39 years of age were 40.6 mg/dL lower (*p* = 0.011) than those without diabetes in the same age group.

The effect of kidney dysfunction on urinary creatinine concentration was not the same across racial/ethnic groups. Non-Hispanic whites with kidney dysfunction had urinary creatinine levels 10.7 mg/dL (*p* = 0.0047) higher than those without kidney disease, whereas the levels for Mexican Americans with kidney disease were 15.5 mg/dL (*p* = 0.0329) lower than those without.

So that we could include children and adolescents in our analyses, we next performed multiple linear regression analyses that included all ages. Subjects ≥20 years of age were only included if they could be classified as not having diabetes and as not having moderately or severely decreased kidney function.

Coefficients from the multiple linear regression model are presented in Table 5. The *R*^2^ of the model was 0.175. Statistically significant categorical independent variables in the model included race/ethnicity, sex, and age group. Neither hyperthyroidism nor hypertension was a significant factor in the model. The continuous independent variable BMI was also a statistically significant factor. Statistically significant interactions were observed between race and age group (*p* = 0.0002) and between sex and age group (*p* < 0.0001).

According to the model results, the effect of age category on urinary creatinine concentrations differed among each racial/ethnic group. Among Mexican Americans, urinary creatinine levels for 20- to 29-year-olds were 44.3 mg/dL higher (*p* < 0.0001) than those for 50- to 59-year-olds. Among non-Hispanic whites, this difference was 55.8 mg/dL (*p* < 0.0001), and among non-Hispanic blacks, 57.5 mg/dL (*p* < 0.0001).

BMI also was significantly related (*p* < 0.0001) to urinary creatinine concentrations. According to the model results, every unit increase in BMI was associated with a 1.30 mg/dL increase in urinary creatinine. Thus, persons with a BMI at the 90th percentile (31.37 kg/m^2^) would be expected to have urinary creatinine levels about 8.6 mg/dL higher than persons of the same demographic group but with a BMI at the median (24.75 kg/m^2^). However, when FFM is included in the model, it interacts strongly with BMI. For example, at the median FFM (2574.97 units), a one-unit increase in BMI results in a 0.92-mg/dL increase in urinary creatinine. At the 75th percentile FFM (2692.15 units), a one-unit increase in BMI is associated with a 0.5-mg/dL increase in urinary creatinine. At the 25th percentile FFM (2462.47 units), a one-unit increase in BMI is associated with a 1.33-mg/dL increase in urinary creatinine. Thus, at higher FFM, BMI has a smaller effect on urinary creatinine.

## Discussion

Biomonitoring of exposure is used in the workplace to evaluate a person’s chemical exposure during the workday and to provide some standard measure for allowable individual workplace exposures. When timed urine excretion (to determine UER) or 24-hr samples are not collected, the chemical measurement is routinely adjusted using creatinine to correct for urine concentration/dilution in spot samples.

For occupational monitoring, the WHO has recommended exclusionary guidelines for urinary creatinine concentrations to identify individual samples that are invalid for chemical analysis. The rationale behind these guidelines is that urine samples with extremely low creatinine concentrations are too dilute and may impair detection of low levels of toxicants, whereas samples with extremely high creatinine concentrations indicate dehydration, which could have changed the kidney’s secretion, excretion, and/or reabsorption of the target chemical. Therefore, analysis of either dilute or concentrated spot samples would not result in an analyte concentration representative of actual exposures. Typical statistical rules of exclusion of outliers would exclude the upper and lower 1 or 5% of the population. However, our data indicate that in some demographic categories, almost no one would be excluded using these criteria. In other demographic categories, as many as 20% of the participants would be excluded. These data support the findings recently reported by Wilder et al. (unpublished data). For example, essentially no Mexican-American female adults ≥70 years of age had urinary creatinine > 300 mg/dL. However, in the same demographic group, about 19% of the samples would be excluded because their urinary creatinine concentrations were < 30 mg/dL.

The WHO guidelines may have been established for occupational monitoring using a workforce with less diversity than the U.S. workforce. If only non-Hispanic white males 20–60 years of age are considered, approximately 10% of the samples would have been excluded, 5% for each exclusionary criterion. Among both sexes in this age range or women alone, approximately 15% of samples would have been excluded, with the majority (9–13%) excluded for being too dilute. In the U.S. population as a whole, samples from nearly 10 million women could be excluded using criteria that were likely not established using data from women. Clearly, with the change in the composition of the modern U.S. workforce to include women, multiple racial/ethnic groups, and older workers because of the increasing retirement age, the guidelines for sample exclusion should be re-evaluated to reflect the results shown in Table 2. In addition, a special reconsideration, or perhaps elimination, of the lower limit of acceptable creatinine concentration should be given. As analytical technology for measuring environmental toxicants or their metabolites in urine samples has dramatically improved over the last several decades, driving the limits of detection very low, detection of chemicals in urine samples considered “dilute” is much less likely to be an issue of concern. Rather, intermittent or low-level exposures will likely have a greater effect on the ability for a given marker of exposure to be measured with current analytical technology.

We observed a small but statistically significant increase in creatinine concentrations in the morning compared with the afternoon and evening. Although we have no information suggesting the morning urine collections in NHANES III were first morning voids, our analyses appear consistent with the general thought that urine from a first morning void is more concentrated.

In the early 1980s, biomonitoring for nonoccupational, environmental exposures became an important exposure assessment tool in epidemiologic studies evaluating environmental exposure risks. In these studies, 24-hr samples were costly and logistically impractical to collect. Therefore, in keeping with the most common approach in workplace monitoring, spot urine samples were collected and chemical measurements were adjusted using creatinine. This approach was generally considered the only valid way to adjust spot urine samples for comparison across groups, even though limited data were available to evaluate the validity of this adjustment. With the increase in the number of child health studies in the 1990s, including assessing *in utero* exposures by analyzing the urine of pregnant women, the variation in creatinine concentrations among different age groups has become increasingly apparent. Several researchers have noted significant differences in chemical exposures among children and adults (Aprea et al. 2000; Heudorf and Angerer 2001; Mills and Zahm 2001; Wilder et al., unpublished data), and most have recognized and reported that creatinine adjustment elevates the urinary chemical concentrations in children compared with adults.

The differences between children and adults are due partly to differences in lean muscle mass. Children and the elderly tend to have less muscle than active adults. Accordingly, children have lower FFM than adults. Because lean muscle produces the vast majority of creatinine in the body, we evaluated the relation between FFM and urinary creatinine. Indeed, FFM and urinary creatinine were significantly associated (*r* = 0.222; *p* < 0.0001); however, the magnitude of their correlation was much lower than expected. When FFM is considered in the linear regression model, it accounts for much, but not all, of the significant associations with age, sex, and race. Because bioimpedance analysis is not performed in most studies collecting biomonitoring data for exposure assessments, age, sex, and race can be used in concert as a surrogate for FFM. Further, because the FFM accounts for a significant proportion of the variation of creatinine, creatinine-adjusted measurements may serve as a useful surrogate for estimating the size-related dose of an individual (Barr et al. 2004).

Urinary biomonitoring measurements are used to assess exposures of individuals and population groups. For an individual, if the urinary chemical level is divided by the creatinine concentration to adjust for dilution, one must recognize that the urinary creatinine concentration varies by age, sex, and race/ethnicity (Mage et al. 2004). Therefore, it would be best for “normal” or “reference” ranges for creatinine-adjusted urinary levels to be available for separate demographic groups, (e.g., children, adolescents, and adults), rather than just for the total population. The *Second National Report on Human Exposure to Environmental Chemicals* (National Center for Environmental Health 2003) provides separate reference ranges for 116 chemicals by age, sex, and race/ethnicity. In addition, the report provides reference ranges for non-creatinine-adjusted levels.

For population groups, public health scientists use the creatinine-adjusted urinary chemical level in two types of models. In model 1, the creatinine-adjusted urinary chemical level is a dependent variable, and other variables are regressed against it to determine significant predictors of exposure to that chemical. In model 2, the creatinine-adjusted urinary chemical level is an independent variable used to determine if that chemical exposure is a significant predictor of a disease outcome. In both models, the urinary chemical concentration is typically divided by the urinary creatinine level, and the resulting concentration, expressed per weight of creatinine, is the variable used.

In model 1, where the creatinine-corrected urinary level is the dependent variable, independent variables may be unrelated to the chemical concentration itself but related to the urinary creatinine concentration. In such a case, the independent variable could potentially achieve statistical significance only because it is related to urinary creatinine. Because age, sex, and race/ethnicity all relate to urinary creatinine, this possibility would have to be considered if they were significant predictors of creatinine-corrected urinary chemical levels.

In model 2, a similar problem could exist in which the creatinine-corrected urinary level may be a significant predictor of a health outcome only because the health outcome is related to urinary creatinine levels, not to the levels of the chemical. This would be a less likely scenario than model 1 but is possible because the urinary level is a ratio of a chemical concentration divided by urinary creatinine concentration.

A straightforward solution to both of these potential problems in interpreting multiple regression results is to separate the urinary chemical concentration from the urinary creatinine concentration in the regression models. For model 1, the dependent variable would be the urinary chemical concentration, unadjusted for creatinine. Urinary creatinine concentration would be included in the multiple regression as an independent variable. In this manner, the urinary chemical concentration is adjusted for urinary creatinine, because urinary creatinine is an independent variable, and other covariates in the model are also adjusted for urinary creatinine. Statistical significance of independent variables would therefore not be due to association with urinary creatinine concentration.

Similarly, in model 2, urinary chemical concentration (unadjusted for creatinine) would be included with urinary creatinine as independent variables to predict the health outcome. The health outcome and the urinary chemical concentration variables are adjusted for creatinine by the urinary creatinine independent variable, so any association of the health outcome with chemical concentration would not be influenced by a relationship with urinary creatinine levels.

The present study has several limitations. First, some of the variables used in our evaluation of the data such as the bioimpedance measurements and serum creatinine measurements were available only for persons > 12 years of age. Second, fasting times may have differed among participants and no dietary variables were considered in the analysis. Third, children < 6 years of age were not evaluated. Fourth, first morning void samples were not targeted for collection, so few were likely present in our study; therefore, these findings may not be directly applicable to first morning void samples. Last, upper-bound confidence intervals could not be established for seven of the 90th-percentile estimates given for creatinine levels in different age, sex, and racial/ethnic demographic groups.

## Conclusions

Generally, in epidemiologic studies it is not practical to collect 24-hr urine samples or, when young children are involved, even first morning voids. Therefore, spot samples are generally the urine samples that are analyzed for assessing human exposures to many chemicals. The urinary concentrations of these chemicals are often reported on a weight/volume basis and a creatinine-adjusted basis. However, urinary creatinine concentrations differ dramatically among different demographic groups; thus, biomonitoring studies using creatinine concentrations to adjust the concentrations of environmental and occupational chemical concentrations should seriously consider the impact these findings will have on the data. For an individual, the creatinine-adjusted concentration of an analyte should be compared with a “reference” range derived from persons in a similar demographic group (e.g., children with children, adults with adults). For multiple regression analysis of population groups, we recommend that the analyte concentration (unadjusted for creatinine) be included in the multiple regression analysis with urinary creatinine added as a separate independent variable. This approach allows the urinary analyte concentration to be appropriately adjusted for urinary creatinine and the statistical significance of other variables in the model (e.g., age, sex, race/ethnicity) to be independent of effects of urinary creatinine concentration.

## Figures and Tables

**Table 1:** Clinical parameters for designation of health status of individuals in NHANES III (1988–1994) survey.

Health status	Clinical parameter
Diabetes*^a^*	Blood glucose > 126 mg/dL after 8-hr fast
Hypertension*^b^*	Systolic value > 140 mm Hg or diastolic > 90 mm Hg
Hyperthyroidism	Serum thyroid-stimulating hormone > 5 μU/mL
Kidney dysfunction	Glomerular filtration rate < 60 mL/min/1.73 m^2^

a Also included individuals who were told by a physician that they had diabetes.

b Also included individuals who were told by one physician two or more time or by two or more physicians that they were hypertensive. Systolic and diastolic measurements were the average of three measurements.

**Table 2:** Weighted quantiles (95% CIs) of urinary creatinine concentrations (mg/dL) in the NHANES III (1988–1994) study population in persons 6–90 years of age.

Race/ethnicity, age (years)	All					Male					Female				
	No.	10th	50th	90th	Mean	No.	10th	50th	90^th^	Mean	No.	10th	50th	90th	Mean
All*^a^*
All	22,245	33.54 (32.07–35.22)	118.6 (115.6–121.4)	237.2 (234.7–241.1)	130.4 (128.2–132.7)	10,610	49.56 (46.08–53.30)	137.2 (134.2–141.0)	254.4 (249.9–262.1)	148.3 (145.3–151.3)	11,635	27.36 (26.04–28.90)	99.49 (97.15–102.9)	217.7 (212.6–224.0)	113.5 (110.7–116.3)
6–11	3,078	42.84 (38.77–46.55)	98.09 (93.81–102.2)	163.1 (157.9–173.4)	102.1 (98.91–105.2)	1,590	49.91 (43.18–55.95)	97.84 (92.79–103.7)	164.7 (158.1–179.6)	104.4 (100.3–108.5)	1,488	33.22 (29.54–40.47)	98.34 (91.40–104.0)	160.6 (153.4–171.7)	99.48 (95.27–103.7)
12–19	3,095	62.14 (56.47–67.04)	150.2 (145.1–158.7)	271.2 (263.0–283.4)	161.5 (156.7–166.2)	1,461	65.27 (60.08–75.83)	151.9 (145.3–163.6)	271.2 (258.8–285.3)	163.6 (157.3–169.9)	1,634	56.04 (46.63–64.40)	149.5 (140.5–158.7)	271.6 (261.3–290.1)	159.3 (153.4–165.1)
20–29	3,438	47.45 (42.53–53.42)	153.8 (147.1–160.7)	275.4 (266.4–294.4)	161.8 (156.6–166.9)	1,608	71.64 (61.62–79.74)	172.8 (161.6–185.3)	297.2 (283.5–324.2)	183.0 (175.4–190.6)	1,830	37.24 (31.64–44.04)	132.8 (126.1–141.6)	246.6 (236.4–264.6)	141.0 (135.0–146.9)
30–39	3,259	31.15 (28.80–36.01)	128.8 (121.5–135.8)	245.7 (239.0–259.1)	138.0 (132.4–143.5)	1,438	44.77 (39.90–55.96)	150.5 (140.2–162.1)	263.3 (251.8–285.2)	157.9 (150.1–165.7)	1,821	27.36 (24.80–29.49)	106.9 (100.6–113.9)	227.7 (215.3–240.0)	118.8 (112.6–125.0)
40–49	2,542	26.32 (23.20–30.79)	119.0 (112.3–124.6)	226.2 (216.4–238.8)	124.6 (120.1–129.1)	1,203	43.24 (33.38–54.56)	146.9 (140.5–154.0)	252.3 (235.8–265.8)	149.7 (143.0–156.4)	1,339	20.49 (17.75–24.31)	89.62 (80.26–96.92)	195.1 (185.0–207.8)	100.6 (95.91–105.3)
50–59	1,823	26.80 (25.02–29.92)	98.43 (92.63–102.9)	206.0 (195.2–217.1)	108.1 (103.8–112.5)	838	39.06 (33.30–47.73)	123.5 (114.4–136.4)	227.7 (216.9–243.5)	131.8 (123.6–139.9)	985	22.54 (20.73–25.22)	73.09 (65.66–81.02)	165.5 (155.0–178.2)	86.06 (80.66–91.46)
60–69	2,243	30.01 (27.67–32.95)	94.22 (89.12–98.97)	193.6 (187.0–200.7)	105.5 (101.8–109.2)	1,134	43.54 (39.06–52.11)	121.4 (114.0–127.0)	213.4 (206.3–231.8)	126.4 (121.2–131.6)	1,109	23.64 (21.53–28.25)	75.37 (69.29–82.09)	167.4 (159.3–179.3)	87.91 (82.50–93.32)
≥70	2,767	29.37 (27.41–31.37)	86.23 (82.36–90.57)	179.6 (175.3–189.1)	97.99 (95.14–100.8)	1,338	43.77 (39.62–50.19)	107.4 (103.0–115.4)	199.2 (188.6–210.9)	117.5 (112.8–122.2)	1,429	23.90 (21.86–26.68)	69.14 (65.23–74.63)	166.5 (157.2–180.0)	84.51 (80.87–88.15)
Non-Hispanic white															
All	8,150	30.94 (29.31–33.10)	112.7 (109.7–115.9)	229.5 (224.7–236.1)	124.6 (122.0–127.2)	3,820	45.91 (41.88–50.56)	133.0 (129.6–137.2)	249.2 (242.3–259.3)	144.0 (140.3–147.8)	4,330	25.27 (24.03–26.63)	92.09 (87.71–96.48)	205.9 (200.5–212.9)	106.1 (103.2–109.0)
6–11	800	42.75 (38.08–47.69)	98.11 (92.57–103.8)	155.1 (149.0–166.7)	99.92 (95.85–104.0)	413	48.74 (42.61–59.12)	97.19 (90.56–103.9)	158.6 (149.1–169.9)	102.1 (96.98–107.3)	387	32.95 (28.89–40.18)	99.00 (90.86–107.6)	152.0 (145.5–169.4)	97.48 (92.8–102.16)
12–19	790	55.90 (50.31–63.98)	147.1 (139.2–156.0)	261.4 (248.8–278.3)	156.0 (149.8–162.1)	348	61.84 (55.72–74.85)	147.4 (138.3–159.2)	252.0 (237.8–275.1)	155.7 (147.8–163.6)	442	47.86 (39.55–61.86)	145.3 (135.6–156.6)	269.6 (252.1–301.7)	156.2 (147.5–164.9)
20–29	879	42.72 (36.40–50.15)	143.3 (137.1–155.0)	271.7 (258.8–296.4)	154.8 (148.2–161.4)	388	63.81 (53.31–79.41)	169.7 (159.6–185.2)	299.4 (281.2–333.8)	181.7 (171.4–192.1)	491	31.55 (26.10–40.42)	120.3 (110.2–132.1)	233.7 (214.7–246.5)	128.9 (121.9–136.0)
30–39	1,025	30.03 (27.68–34.07)	123.3 (115.2–131.9)	237.0 (231.2–254.5)	133.3 (126.5–140.1)	437	42.21 (36.31–53.47)	146.3 (132.9–162.3)	252.5 (241.1–286.5)	153.7 (143.4–163.9)	588	25.83 (23.08–29.71)	103.3 (94.32–108.3)	221.3 (208.0–232.5)	113.2 (106.4–120.0)
40–49	893	23.40 (20.58–28.72)	113.9 (106.6–120.2)	219.2 (208.7–234.9)	119.8 (115.0–124.6)	422	37.14 (27.78–49.52)	142.3 (136.0–152.0)	244.5 (225.5–260.1)	145.0 (137.2–152.9)	471	18.61 (16.70–22.96)	78.56 (71.33–95.15)	182.9 (172.8–220.2)	94.46 (89.08–99.83)
50–59	884	26.06 (23.96–28.89)	93.95 (87.35–100.4)	203.3 (186.8–218.4)	104.3 (98.83–109.8)	409	37.74 (32.01–46.84)	117.6 (107.5–133.7)	226.0 (213.8–244.7)	129.0 (118.3–139.6)	475	22.64 (20.46–25.30)	70.44 (60.83–77.02)	154.8 (144.1–170.3)	81.36 (75.52–87.20)
60–69	963	30.04 (27.35–33.20)	90.41 (84.88–97.18)	189.0 (184.0–197.1)	102.7 (98.60–106.8)	495	43.84 (39.83–54.25)	121.0 (110.5–125.2)	210.3 (201.7–231.6)	124.8 (119.4–130.2)	468	22.76 (20.24–27.22)	72.55 (64.84–80.14)	162.9 (153.6–174.0)	83.35 (77.53–89.17)
≥70	1,916	28.69 (26.60–30.86)	84.89 (79.77–88.55)	176.9 (171.2–187.6)	96.03 (92.84–99.22)	908	42.90 (38.70–49.49)	107.0 (101.1–114.9)	196.4 (184.3–209.6)	116.2 (111.0–121.3)	1,008	23.44 (21.27–27.17)	66.73 (63.62–72.34)	160.9 (152.0–173.8)	82.30 (78.16–86.44)
Non-Hispanic black															
All	6,664	57.24 (54.37–61.00)	153.3 (149.6–158.1)	282.6 (277.7–289.5)	165.4 (162.3–168.5)	3,117	72.84 (68.31–76.59)	170.3 (164.5–177.6)	298.5 (292.7–310.3)	181.9 (177.3–186.4)	3,547	49.64 (45.94–53.27)	140.1 (136.5–144.4)	265.1 (257.6–272.6)	151.3 (147.8–154.8)
6–11	1,060	53.72 (47.81–58.87)	113.8 (110.1–120.9)	201.2 (192.5–211.3)	120.9 (116.3–125.6)	553	54.58 (47.09–60.66)	113.2 (107.4–120.6)	199.5 (188.2–209.9)	120.3 (114.8–125.8)	507	52.64 (44.80–59.58)	115.6 (108.8–122.1)	203.9 (192.5–215.0)	121.6 (115.7–127.5)
12–19	1,113	83.12 (74.04–92.62)	179.4 (172.1–187.3)	310.9 (302.2–325.6)	193.1 (185.4–200.8)	530	88.38 (76.57–102.1)	188.5 (179.9–200.5)	322.3 (313.1–343.1)	203.9 (193.8–214.0)	583	75.86 (65.30–87.54)	172.3 (163.0–182.3)	295.0 (279.4–317.6)	182.4 (173.6–191.2)
20–29	1,098	82.04 (69.40–93.87)	193.4 (188.1–202.9)	315.0 (301.5–332.9)	200.1 (192.8–207.5)	484	90.23 (76.67–115.3)	207.0 (193.1–224.3)	339.9 (316.1–377.4)	214.7 (202.5–227.0)	614	77.77 (61.32–89.35)	185.2 (175.2–194.3)	292.9 (285.9–315.4)	188.0 (179.6–196.2)
30–39	1,120	64.14 (59.23–68.77)	164.4 (155.0–173.6)	284.9 (272.8–299.6)	172.0 (165.7–178.3)	480	82.70 (72.56–97.43)	186.1 (176.9–197.6)	312.0 (290.2–326.5)	193.1 (184.2–202.1)	640	56.48 (48.35–63.58)	148.7 (140.9–157.0)	267.4 (252.3–283.1)	155.3 (146.8–163.9)
40–49	798	53.76 (47.98–65.24)	152.8 (140.8–169.3)	275.2 (260.6–288.1)	164.2 (155.8–172.6)	359	78.50 (68.15–92.70)	180.6 (171.0–192.2)	293.5 (279.4–321.4)	189.5 (181.2–197.7)	439	44.54 (36.66–53.85)	130.0 (119.9–146.6)	238.3 (226.1–267.4)	142.9 (132.2–153.7)
50–59	475	35.78 (28.68–48.23)	134.6 (118.0–150.0)	245.3 (228.4–264.5)	164.2 (155.8–172.6)	210	67.50 (57.98–81.14)	165.3 (151.2–174.7)	269.7 (242.4–NE)	169.0 (157.8–180.1)	265	26.01 (22.35–36.53)	111.0 (95.86–125.3)	217.8 (195.0–232.7)	117.2 (105.9–116.7)
60–69	557	47.22 (41.17–54.59)	115.9 (107.1–126.2)	224.5 (210.3–242.4)	140.0 (130.7–149.3)	279	62.87 (49.25–75.93)	150.1 (139.9–162.2)	270.2 (245.7–288.8)	158.6 (149.3–167.9)	278	41.38 (36.32–50.60)	96.15 (90.57–103.2)	186.4 (171.7–207.9)	108.8 (100.8–116.7)
≥70	443	38.79 (34.55–46.27)	110.9 (105.6–120.3)	209.8 (203.8–224.2)	129.3 (122.4–136.3)	222	49.05 (40.90–56.87)	130.0 (11.2–145.9)	220.8 (204.7–NE)	136.0 (126.7–145.4)	221	34.58 (30.28–42.77)	104.1 (93.44–109.7)	203.4 (185.0–NE)	112.2 (103.5–120.9)
Mexican American															
All	6,496	38.35 (35.69–42.13)	123.3 (120.2–126.4)	236.5 (231.6–243.7)	132.9 (129.7–136.1)	3,253	50.52 (45.74–55.94)	138.2 (133.2–144.5)	252.5 (245.1–264.9)	147.2 (142.4–151.9)	3,243	30.80 (28.08–34.80)	106.0 (102.7–110.1)	218.3 (211.2–224.9)	117.6 (114.3–120.9)
6–11	1,083	31.92 (26.14–37.65)	87.99 (82.28–92.45)	154.4 (142.6–166.6)	92.24 (87.67–96.82)	548	32.22 (25.61–41.30)	89.53 (84.26–97.65)	160.3 (144.3–173.5)	94.76 (89.59–99.93)	535	30.10 (24.10–38.54)	85.55 (77.88–95.20)	152.2 (135.4–165.8)	89.57 (82.58–96.55)
12–19	1,039	57.50 (50.37–35.25)	140.0 (134.9–145.8)	249.0 (236.3–265.6)	148.2 (142.1–154.3)	518	57.72 (47.04–70.12)	142.3 (133.6–152.3)	255.7 (237.5–275.7)	151.5 (141.3–161.6)	521	56.75 (46.27–65.77)	133.6 (127.5–145.7)	240.4 (226.5–262.8)	144.8 (138.4–151.3)
20–29	1,311	50.96 (42.23–61.01)	148.9 (142.8–156.9)	261.9 (247.8–282.7)	155.4 (150.2–160.7)	664	65.14 (52.75–77.76)	166.9 (157.8–174.4)	276.3 (258.8–297.8)	168.9 (162.4–175.4)	647	38.47 (33.15–48.69)	126.9 (117.1–137.6)	246.5 (230.1–269.9)	138.5 (132.0–145.0)
30–39	979	36.71 (32.00–44.12)	132.4 (126.9–138.5)	251.8 (236.7–265.9)	139.9 (133.5–146.3)	464	52.12 (45.63–63.38)	152.2 (143.9–159.7)	270.9 (259.3–285.2)	160.9 (153.1–168.6)	515	29.57 (25.79–33.52)	108.8 (101.0–115.5)	216.1 (189.2–236.6)	116.7 (108.9–124.6)
40–49	738	36.33 (31.16–45.31)	126.5 (118.7–136.6)	227.7 (217.3–240.6)	133.0 (126.6–139.3)	376	53.58 (45.62–66.69)	146.3 (138.0–157.9)	244.7 (231.8–263.5)	153.6 (146.1–161.1)	362	30.05 (23.07–40.38)	105.3 (90.92–120.9)	202.8 (190.3–216.5)	111.2 (101.6–120.7)
50–59	367	27.21 (22.37–33.48)	99.12 (85.91–113.7)	196.2 (188.0–210.9)	109.1 (100.2–118.1)	177	46.38 (37.21–60.52)	125.2 (106.7–151.5)	218.5 (202.3–NE)	134.5 (122.6–146.4)	190	18.60 (15.41–26.66)	71.09 (58.38–91.33)	170.7 (153.9–183.6)	85.27 (76.36–94.18)
60–69	641	21.39 (19.67–27.51)	88.47 (79.08–97.59)	196.8 (184.4–211.1)	99.68 (94.73–104.6)	326	28.99 (19.82–47.54)	111.2 (102.1–130.0)	201.6 (174.7–217.9)	116.9 (108.4–125.3)	315	20.89 (14.85–25.71)	66.85 (57.74–80.61)	192.2 (157.1–NE)	86.36 (77.82–94.90)
≥70	338	27.77 (21.93–35.03)	94.66 (87.62–104.8)	174.8 (160.4–201.0)	99.46 (91.72–107.2)	180	59.55 (50.02–70.02)	116.8 (101.7–135.7)	190.8 (175.7–NE)	123.8 (114.3–133.3)	158	20.84 (16.97–29.49)	67.79 (55.72–81.33)	145.7 (116.5–NE)	76.47 (66.0–86.94)

NE, could not be reliably estimated.1

a All population data, including those individuals not grouped into one of the three race/ethnicity categories, are presented.

**Table 3:** Percentage of each demographic group in NHANES III (1988–1994) whose urinary creatinine concentrations (mg/dL) fell outside the WHO guideline range (i.e., < 30 mg/dL or > 300 mg/dL).

	All			Male			Female		
Race/ethnicity, age(years)	No.	< 30 mg/dL	> 300 mg/dL	No.	< 30 mg/dL	> 300 mg/dL	No.	< 30 mg/dL	> 300 mg/dL
All									
All	22,245	7.7	3.3	10,610	4.0	4.6	11,635	11	2.2
6–11	3,078	4.7	0.1	1,590	2.9	0.1	1,488	6.7	0.1
12–19	3,095	2.3	6.5	1,461	1.6	6.0	1,634	3.1	7.0
20–29	3,438	5.2	6.9	1,608	3.4	10	1,830	7.0	4.2
30–39	3,259	8.4	4.2	1,438	4.3	6.4	1,821	12	2.0
40–49	2,542	11	2.5	1,203	5.9	3.8	1,339	16	1.3
50–59	1,823	12	0.9	838	6.0	1.5	985	17	0.3
60–69	2,243	9.3	0.6	1,134	3.9	1.2	1,109	14	0.1
≥70	2,767	10	0.7	1,338	3.5	1.1	1,429	15	0.5
Non-Hispanic white									
All	8,150	8.8	3.0	3,820	4.5	4.2	4,330	13	1.8
6–11	800	4.3	0.0	413	2.6	0.0	387	6.1	0.0
12–19	790	3.0	6.1	348	2.0	4.6	442	3.9	7.6
20–29	879	6.2	6.4	388	3.9	10	491	8.4	3.0
30–39	1,025	9.2	4.0	437	4.9	6.2	588	14	1.8
40–49	893	13	2.3	422	7.0	3.5	471	19	1.1
50–59	884	13	0.6	409	7.4	1.1	475	18	0.2
60–69	963	9.3	0.4	495	3.0	0.8	468	15	0.0
≥70	1,916	11	0.8	908	3.6	1.2	1,008	15	0.5
Non-Hispanic black									
All	6,664	2.8	7.1	3,117	1.5	9.8	3,547	3.8	4.8
6–11	1,060	3.4	0.6	553	2.7	0.4	507	4.2	0.8
12–19	1,113	0.6	12	530	0.2	15	583	1.1	8.5
20–29	1,098	1.7	13	484	1.6	17	614	1.7	9.5
30–39	1,120	2.8	7.6	480	1.8	12	640	3.5	4.5
40–49	798	3.3	5.8	359	1.4	8.2	439	4.9	3.7
50–59	475	6.9	3.5	210	1.1	5.8	265	12	1.6
60–69	557	2.4	2.6	279	1.0	54	278	3.4	0.7
≥70	443	4.9	1.1	222	3.6	1.2	221	5.9	0.6
Mexican American									
All	6,496	6.5	3.1	3,253	4.4	4.3	3,243	8.8	1.8
6–11	1,083	8.9	0	548	8.0	0.0	535	9.8	0.0
12–19	1,039	2.8	4.2	518	2.0	5.0	521	3.5	3.4
20–29	1,311	4.8	5.4	664	3.8	6.5	647	6.1	3.9
30–39	979	6.7	3.5	464	3.9	5.4	515	9.7	1.4
40–49	738	6.5	2.1	376	4.0	4.0	362	9.2	0.2
50–59	367	10	1.5	177	3.3	3.3	190	16	0.0
60–69	641	15	0.3	326	10	0.8	315	19	0.0
≥70	338	11	0.0	180	2.8	0.0	158	19	0.0

**Table 4:** Weighted mean urinary creatinine concentration (mg/dL) for each collection time frame during the day.

Collection time frame	No.	Mean creatinine (mg/dL)	Contrasted to morning	Contrasted to afternoon	Contrasted to evening
Morning	10,621	133.5	NA	*p* = 0.058	*p* = 0.001
Afternoon	7,190	128.6	*p* = 0.058	NA	*p* = 0.27
Evening	4,434	126.1	*p* = 0.001	*p* = 0.27	NA

NA, not applicable. The concentrations were corrected for age, race/ethnicity, sex, and BMI. Each mean was contrasted to the means of other collection time frames using an analysis of covariance test to determine whether they were statistically different.

**Table 5:** Coefficients of the independent variables from the multiple linear regression model of urinary creatinine concentrations (dependent variable).

	Independent variable	
Variable	Coefficient ± SE	*p*-Value
Intercept	53.51 ± 6.83	< 0.0001
Race/ethnicity		
Non-Hispanic white (1)	−7.33 ± 5.00	0.1486
Non-Hispanic black (2)	20.82 ± 5.68	0.0006
Mexican American (3)	0.00 ± 0.00	NA
Sex		
Male (1)	34.59 ± 4.14	< 0.0001
Female (2)	0.00 ± 0.00	NA
Age group (years)		
6–11 (1)	12.55 ± 5.24	0.0026
12–19 (2)	62.90 ± 5.64	< 0.0001
20–29 (3)	43.56 ± 5.70	< 0.0001
30–39 (4)	29.78 ± 5.78	< 0.0001
40–49 (5)	16.65 ± 6.42	0.0125
50–59 (6)	−1.17 ± 6.24	0.8524
60–69 (7)	−8.47 ± 4.81	0.0847
≥70 (8)	0.00 ± 0.00	NA
BMI (continuous)	1.30 ± 0.19	< 0.0001
Race/ethnicity × age group		
(1) × (1)	16.19 ± 6.09	0.0106
(1) × (2)	16.14 ± 6.67	0.0192
(1) × (3)	10.74 ± 6.68	0.1141
(1) × (4)	4.34 ± 5.66	0.4469
(1) × (5)	−2.40 ± 6.94	0.7308
(1) × (6)	−0.82 ± 5.73	0.8864
(1) × (7)	6.99 ± 4.86	0.1569
(1) × (8)	0.00 ± 0.00	NA
(2) × (1)	8.64 ± 6.48	0.1886
(2) × (2)	24.28 ± 6.48	0.0005
(2) × (3)	28.19 ± 6.50	0.0001
(2) × (4)	15.01 ± 7.12	0.0403
(2) × (5)	14.69 ± 7.77	0.0648
(2) × (6)	14.98 ± 8.27	0.0762
(2) × (7)	8.58 ± 6.35	0.1826
(2) × (8)	0.00 ± 0.00	NA
(3) × (1)	0.00 ± 0.00	NA
(3) × (2)	0.00 ± 0.00	NA
(3) × (3)	0.00 ± 0.00	NA
(3) × (4)	0.00 ± 0.00	NA
(3) × (5)	0.00 ± 0.00	NA
(3) × (6)	0.00 ± 0.00	NA
(3) × (7)	0.00 ± 0.00	NA
(3) × (8)	0.00 ± 0.00	NA
Sex × age group		
(1) × (1)	−30.64 ± 4.26	< 0.0001
(1) × (2)	−30.44 ± 5.86	< 0.0001
(1) × (3)	11.57 ± 5.30	0.0339
(1) × (4)	6.01 ± 7.16	0.4051
(1) × (5)	15.86 ± 5.53	0.0061
(1) × (6)	12.53 ± 7.57	0.1045
(1) × (7)	9.39 ± 5.51	0.0944
(1) × (8)	0.00 ± 0.00	NA
(2) × (1)	0.00 ± 0.00	NA
(2) × (2)	0.00 ± 0.00	NA
(2) × (3)	0.00 ± 0.00	NA
(2) × (4)	0.00 ± 0.00	NA
(2) × (5)	0.00 ± 0.00	NA
(2) × (6)	0.00 ± 0.00	NA
(2) × (7)	0.00 ± 0.00	NA
(2) × (8)	0.00 ± 0.00	NA

NA, not applicable. Numbers in parentheses correspond to the specific racial/ethnic group, sex, or age group for which the interaction term was derived.
